# The *SsAtg1* Activating Autophagy Is Required for Sclerotia Formation and Pathogenicity in *Sclerotinia sclerotiorum*

**DOI:** 10.3390/jof8121314

**Published:** 2022-12-17

**Authors:** Wenli Jiao, Huilin Yu, Xueting Chen, Kunqin Xiao, Dongmei Jia, Fengting Wang, Yanhua Zhang, Hongyu Pan

**Affiliations:** College of Plant Sciences, Jilin University, Changchun 130012, China

**Keywords:** *Sclerotinia sclerotiorum*, transcriptome, *SsAtg1*, sclerotia, autophagy, pathogenicity

## Abstract

*Sclerotinia sclerotiorum* is a necrotrophic phytopathogenic fungus that produces sclerotia. Sclerotia are essential components of the survival and disease cycle of this devastating pathogen. In this study, we analyzed comparative transcriptomics of hyphae and sclerotia. A total of 1959 differentially expressed genes, 919 down-regulated and 1040 up-regulated, were identified. Transcriptomes data provide the possibility to precisely comprehend the sclerotia development. We further analyzed the differentially expressed genes (DEGs) in sclerotia to explore the molecular mechanism of sclerotia development, which include ribosome biogenesis and translation, melanin biosynthesis, autophagy and reactivate oxygen metabolism. Among these, the autophagy-related gene *SsAtg1* was up-regulated in sclerotia. Atg1 homologs play critical roles in autophagy, a ubiquitous and evolutionarily highly conserved cellular mechanism for turnover of intracellular materials in eukaryotes. Therefore, we investigated the function of *SsAtg1* to explore the function of the autophagy pathway in *S. sclerotiorum*. Deficiency of *SsAtg1* inhibited autophagosome accumulation in the vacuoles of nitrogen-starved cells. Notably, Δ*SsAtg1* was unable to form sclerotia and displayed defects in vegetative growth under conditions of nutrient restriction. Furthermore, the development and penetration of the compound appressoria in Δ*SsAtg1* was abnormal. Pathogenicity analysis showed that *SsAtg1* was required for full virulence of *S. sclerotiorum*. Taken together, these results indicate that *SsAtg1* is a core autophagy-related gene that has vital functions in nutrient utilization, sclerotia development and pathogenicity in *S. sclerotiorum*.

## 1. Introduction

*Sclerotinia sclerotiorum* (Lib.) de Bary, a homothallic necrotrophic type phytopathogenic fungus, has an extensive host range [1,2]. Multicellular development is a significant characteristic of *S. sclerotiorum*, which contain sclerotia, compound appressoria and apothecia [3,4]. Sclerotia as a central stage of the life and infection cycle of *S. sclerotiorum*, is not only the long-term survival structure, but also an indispensable part of asexual and sexual reproduction [5,6]. The formation of sclerotia is subject to complex and exquisite regulation, including the cAMP/PKA pathway, MAPK pathway and autophagy [7,8,9,10].

Autophagy is a ubiquitous and evolutionarily highly conserved cellular reaction mechanism for turnover of intracellular materials in eukaryotes [11,12]. During the process of growth and differentiation, in the face of external environmental stress such as nutrient deficiency and hypoxia, cells initiate autophagy to degrade organelles to provide basic energy for cell survival [11,13,14,15]. In filamentous fungi, autophagy is correlated with nutrient balance, secondary metabolism, cell differentiation and pathogenicity [11,13,16]. MoAtg8 interaction with MoAtg4 determines the cell differentiation and pathogenicity of *Magnaporthe oryzae*, meanwhile, loss of *MoAtg8* leads to the obstruction of sexual reproduction and delayed perithecium formation [17]. In *Botrytis cinerea*, the autophagy pathway was blocked after deletion of *BcAtg3*, *BcAtg7* and *BcAtg8* genes, and the mycelial growth, sclerotia formation and virulence of the mutants were significantly defective [18,19]. *FgAtg15* encodes a lipolytic enzyme that is involved in many developmental processes of *Fusarium graminearum*. The virulence of the *FgAtg15* mutant was decreased, and the production of DON toxin by the *FgAtg15* mutant was markedly lower than the wild type [20].

The autophagy related protein Atg1 belongs to the serine/threonine protein kinase family and is the unique protein kinase among yeast autophagy-related proteins. The ATG1-ATG13-ATG17 complex with regulatory subunit Atg13 and scaffold protein Atg17 at the initiation stage of autophagy is recruited to autophagosomes at PAS (pre-autophagosomal structure) adjacent to vacuoles to form related proteins [21,22]. Deletion of *MoAtg1* in *M. oryzae* leads to defects including loss of virulence, reduced sporulation, reduced conidial lipid droplets, malformation and inadequate turgor pressure of appressorium [11]. In *B. cinerea, BcATG1* deficiency inhibited the accumulation of autophagosomes induced by nitrogen starvation and significantly impaired vegetative growth, conidia and sclerotia formation [23]. *FgATG1* affects sporulation and pathogenicity of *F. graminearum* [24]. Atg1, as a core gene that induces autophagy, can coordinate complex signaling pathways to precisely regulate autophagosome formation and plays a crucial role in response to stress and other biological pathways [25]. The Atg1 complex can respond to the TOR kinase. In *M. Oryzae*, the TOR kinase was inhibited under starvation and stress, leading to Atg1 phosphorylation and Atg13 dephosphorylation, forming the Atg1 complex and inducing autophagy [11,16]. Although MAPK and cAMP/PKA pathways have been substantiated to have significant influence in sclerotia formation and pathogenicity of *S. sclerotiorum* [7,26,27,28], the biological function of the autophagy-related protein Atg1 in *S. sclerotiorum* has not been studied.

In this research, RNA sequencing was used to investigate the transcriptomic dynamics of sclerotia development, DEGs at the sclerotial stage were utilized to investigate the molecular mechanism of sclerotia development, which include ribosomal biogenesis, melanin biosynthesis, cell wall/membrane/envelope biogenesis, autophagy and ubiquitination, reactivate oxygen metabolism and signal transduction. Moreover, we identified Atg1, a serine/threonine kinase homolog in *S. sclerotiorum* designated SsAtg1 and further investigated the function of SsAtg1. After deletion of *SsAtg1*, autophagosomes could not be induced. *SsAtg1* is indispensable for sclerotia formation and full virulence of *S. sclerotiorum*. These results demonstrate that the autophagy pathway plays a necessary role in the asexual reproduction and pathogenicity in this necrotrophic fungus.

## 2. Materials and Methods

### 2.1. Strains and Culture Condition

*Sclerotinia sclerotiorum* UF-1 used as the wild-type strain in this study was provided by J. Rollins, University of Florida. ∆*SsAtg1* and ∆*SsAtg1*-c (complemented strain) were grown on PDA (200 g potato, 20 g glucose, 15 g agar per liter) at 25 °C [29].

### 2.2. Gene Functional Annotation and Differential Gene Expression Analysis

Based on sequencing by synthesis (SBS) technology, Illumina HiSeq high-throughput sequencing platform was used to sequence cDNA libraries and produced a large amount of high-quality data (BioMarker). A fold change of ≥2 and a false discovery rate (FDR) < 0.01 were used as the criteria for identifying differentially expressed genes (DEGs). Gene functions were annotated by KEGG (Kyoto Encyclopedia of Genes and Genomes) [30], GO (Gene Ontology) [31], Swiss-Prot [32] and Pfam (Protein family) [33].

### 2.3. Identification and Sequence Information of SsAtg1

The Atg1 phylogenetic tree was constructed by neighbor-joining method with 1000 bootstrap replicates in MEGA7. Conserved domains of SsAtg1 sequence were analyzed by NCBI (https://www.ncbi.nlm.nih.gov/, accessed on 18 February 2022) and Interpro (http://www.ebi.ac.uk/interpro, accessed on 18 February 2022), and distribution and visualization of domains performed by GPS [9].

### 2.4. Plasmid Constructs and Transformation

We used split-marker PCR [34] to obtain gene knockout fragments: nucleotide sequences FR1 and FR2 were amplified by SsAtg1FP1/SsAtg1RP1 and SsAtg1FP2/SsAtg1RP2, respectively. The hygromycin resistance genes were amplified from the containing pUCATPH vector to obtain overlapping HY and YG fragments using M13R/NLC37 and M13F/NLC38 [34]. Then, FR1/HY and FR2/YG were used as templates fused by SsAtg1 FP1/NLC37 and SsAtg1RP2/NLC38 to obtain final knockout fragments. Plasmid pYF11 was used for the overexpression of *SsAtg1*. The PEG-mediated transformation method was used to transform the two sequences into the protoplasts of *S. sclerotiorum*, which was performed as previous described [35,36] with some modification: composition of enzyme solution: NaCl (0.7 M 15 mL), cellulase (150 mg) (Solarbio Cellulase R-10 C8260), Driselase (100 mg) (Sigma D9515). Transformants were purified by using hygromycin (100 μg/mL) and geneticin (100 μg/mL) hyphal-tip selected at least five times, respectively. Information on primers can be found in Table S10.

### 2.5. Stress Treatment

The cell integrity of strains was evaluated by growth on PDA with Congo Red (CR, 0.5 mg/mL), Calcofluor white (CFW, 10 μg/mL) and 0.001% sodium dodecyl sulphate (SDS). To analyze hypersensitivity to ROS, strains were inoculated on PDA supplemented with different concentration of H_2_O_2_ (5, 10 mM). For exploring nutrient uptake, strains were grown on CM (0.2 g KH_2_PO_4_, 0.25 g MgSO_4_·7H_2_O, 0.15 g NaCl, 1 g Ca(NO_3_)_2_·4H_2_O, 10 g glucose, 1 g yeast extract, 1 g casein hydrolysate, 15 g agar per liter), MM (CM lacking 1 g yeast extract and 1 g casein hydrolysate), MM-N (remove Ca(NO_3_)_2_·4H_2_O from the MM), MM-C (MM lacking glucose) and MM-P (MM lacking KH_2_PO_4_). 

### 2.6. Analysis of Compound Appressoria, Sclerotia Formation and Pathogenicity

Glass slides (hydrophobic surface) and onion epidermal peels were used as two different substrata to compare the formation of compound appressoria between WT, ∆*SsAtg1* and ∆*SsAtg1-c*. Image J software was used to quantify the compound appressoria formed on glass slides. Lactophenol cotton blue solution (Sigma) was used to stain the compound appressoria on the onion epidermis, but the invasive hyphae that penetrated onion epidermis did not stain. Sclerotia development was photographed and counted four weeks after culture. To analyze pathogenicity, mycelia-colonized plugs (d = 0.5 mm) were inoculated on soybean leaves and tobacco (*Nicotiana benthamiana*), the lesion areas on leaves were measured after 48 h by image J. Experiments were conducted three times.

### 2.7. Quantitative Real-Time PCR Analysis

As ∆*SsAtg1* does not produce sclerotia, in the process of exploring sclerotia development, we obtained mycelia samples of ∆*SsAtg1* parallel to the time point of appearance of WT sclerotia or melanized sclerotia. Mycelia and sclerotia samples of wild type and ∆*SsAtg1* were obtained, respectively, at the same time. TransZol Up Plus RNA Kit (TransGen Biotech, Beijing, China) was used for total RNA extraction after freezing and grinding in liquid nitrogen. EasyScript All-In-one First-strand cDNA Synthesis SuperMix for Qpcr (One-step Gdna Removal) (TransGen Biotech, Beijing) was used to synthesize cDNA. Gene expression was measured by TransStart Green Qpcr SuperMix (TransGen Biotech, Beijing). NCBI primer was used to design a primer which was used to measure gene expression levels, and actin was used as internal reference. 

### 2.8. Autophagy Assay

To visualize the autophagic process in *S. sclerotiorum*, monodansylcadaverine (MDC) was used as a fluorescent pigment to detect specific markers of autophagosome formation. The autophagosomes were observed in the mycelia of WT and Δ*ssAtg1* cultured in liquid CM at 25 °C for 24 h, then transferred to liquid MM-N for 6 h, the monodansylcadaverine (MDC) kit (Beijing Solarbio Science & Technology Co., Ltd., Beijing, China) was used to stain autophagosome, and then viewed under epi-fluorescence microscopy.

## 3. Results

### 3.1. Transcriptome Analysis Reveals Melanin, Reactive Oxygen and Autophagy Are Associated with Sclerotia Development in S. sclerotiorum

**Figure 1:**
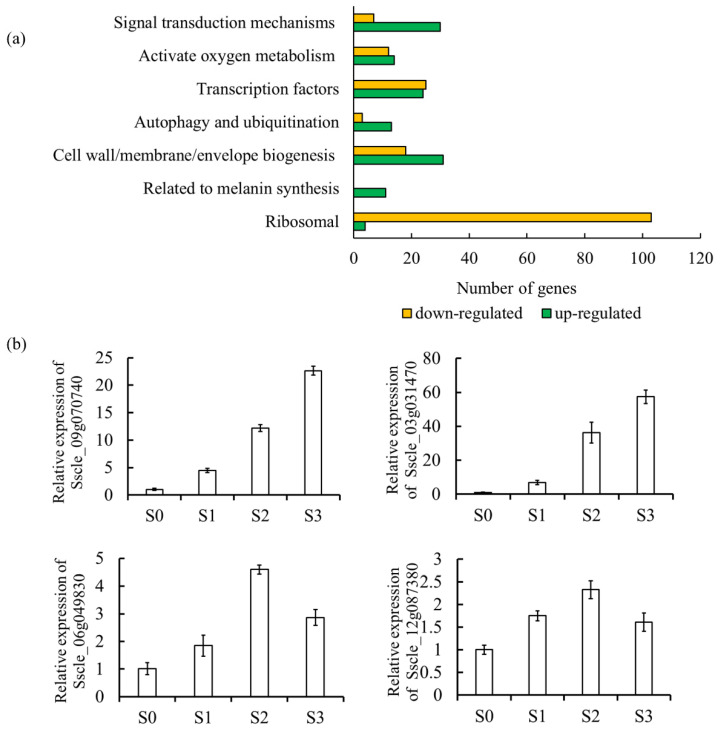
DEGs between hypha and sclerotia in *S. sclerotiorum*. (**a**) GO annotation enrichment in sclerotia relative to hyphae. (**b**) Relative expression of genes in sclerotia at different stages verified by RT-qPCR. Samples of wild-type hyphae (S0, 2d), initiation of sclerotia (S1, 3d), developing sclerotia (S2, 5d) and mature sclerotia (S3, 9d) were collected. Genes *Sscle_09g070740*, *Sscle_03g031470*, *Sscle_06g049830* and *Sscle_12g087380* were selected to assess transcript accumulation dynamics.

In order to explore the mechanism of sclerotia formation, we analyzed differentially expressed genes in hyphae and sclerotia by transcriptomic analysis. In total, 1959 differentially expressed genes (DEGs) were obtained, of which 1040 were up-regulated and 919 were down-regulated. COG (clusters of orthologous groups) was used to annotate 1125 functional genes (Table S1), and GO annotation was used to annotate functional genes within three branches of biological process (1043), cellular component (694) and molecular function (1036) (Figure S1a,b, Tables S1 and S2). Ribosome biogenesis, melanin synthesis, cell wall/membrane/envelope biogenesis, autophagy and ubiquitination, transcription factor, reactivate oxygen metabolism and signal transduction mechanisms were vital biological functions, which were all annotated in DEGs (Figure 1a). The genes with differential expression levels were selected for verification and found to have similar expression trends as those in the transcriptome, indicating that the transcriptome data results were reliable and could be further analyzed (Figure 1b).

#### 3.1.1. Melanin Plays an Important Role in Sclerotia Development

Melanin confers resistance to sclerotia under adverse climatic and soil conditions. Melanogenesis of dihydroxynaphthalene (DHN) is synthesized through the pentaketide pathway, which has also been verified in *S. sclerotiorum* [37,38]. Scytalone dehydratases (*Sscle_03g031470*), trihydroxynaphthalene reductase (*Sscle_09g070740*) and laccase (*Sscle_02g018680*, *Sscle_02g021570*, *Sscle_12g090390*), which contribute to melanin biosynthesis, were remarkably increased in the sclerotia stage (Table S3). 

#### 3.1.2. Cell Wall/Membrane/Envelope Biogenesis

The formation of sclerotia is accompanied by cell wall thickening. In the transcriptome analysis, it was found that the expression of genes related to the formation of the cell wall increased in the sclerotia stage including chitin synthase (*Sscle_14g099740*, *Sscle_02g018150*) and glucan 1,3-beta-glucosidase (*Sscle_01g002030*). In contrast, the expression levels of genes related to cell membrane structure were mostly decreased in the sclerotia stage including ergosterol synthesis genes *ERG6 (Sscle_10g080660*, *Sscle_11g085920*, *Sscle_07g056400*), *ERG3* (*Sscle_02g021190*, *Sscle_14g100930*) and *ERG24* (*Sscle_06g051840*). Glutamine-fructose 6-phosphate aminotransferase (*Sscle_02g019730*), a rate-limiting enzyme in the synthesis of uridine 5 ‘-diphospho-N-acetyl glucosamine (UDP-GlCNAC), also decreased in the sclerotia stage (Table S4).

#### 3.1.3. Ribosome Biogenesis and Translation

Ribosomes, composed of ribosomal RNA and dozens of different ribosomal proteins, are molecular machines for protein synthesis in cells. In the sclerotia stage, 103 genes related to ribosome structure, function and synthesis were down-regulated, indicating that ribosome activities, such as mRNA translation and protein folding, were reduced in the sclerotia stage (Tables S5 and S6).

#### 3.1.4. Reactive Oxygen Metabolism

Catalase and superoxide dismutase are important intracellular enzymes that metabolize reactive oxygen species. Superoxide dismutase catalyzes the disproportionation of superoxide anion radical (O_2_^−^) to H_2_O_2_ and O_2_, while catalase catalyzes the decomposition of H_2_O_2_ into O_2_ and H_2_O. The increased expression levels of catalase (*Sscle_01g011560*, *Sscle_04g037170*, *Sscle_15g104430*) and superoxide dismutase (*Sscle_05g043700*) encoding genes suggests that a large amount of reactive oxygen species may be produced in the sclerotia-forming stage. In contrast, the expression of glutathione reductase (*Sscle_09g070370*), thioredoxin reductase (*Sscle_05g046390*) and NADPH-cytochrome P450 reductase (*Sscle_10g078540*, *Sscle_07g060800*) were decreased in the sclerotia-forming stage. These results further indicate that oxidative stress plays an important role in the development of sclerotia (Table S7).

#### 3.1.5. Autophagy

Autophagy is a ubiquitous programmed cell degradation mechanism in eukaryotic cells, that can remove and degrade the excess biological macromolecules produced in biological processes and use the degraded substances for energy supply and cell structure reconstruction. The expression of autophagy related genes *SsAtg1* (*Sscle_12g087380*) and *SsAtg13* (*Sscle_15g103100*) were increased in the sclerotia-forming stage (Table S8). As a core gene for autophagy induction, *Atg1* can coordinate complex signaling pathways to precisely regulate autophagosome formation and plays a crucial role in response to stress, growth and development and other biological pathways. The above results provide strong evidence for the important role of the autophagy pathway in sclerotia formation. In order to further explore the mechanism of autophagy on sclerotia development, the biological function of *SsAtg1* was further studied.

### 3.2. SsAtg1 Is a Key Gene Regulating Autophagy in S. sclerotiorum

**Figure 2:**
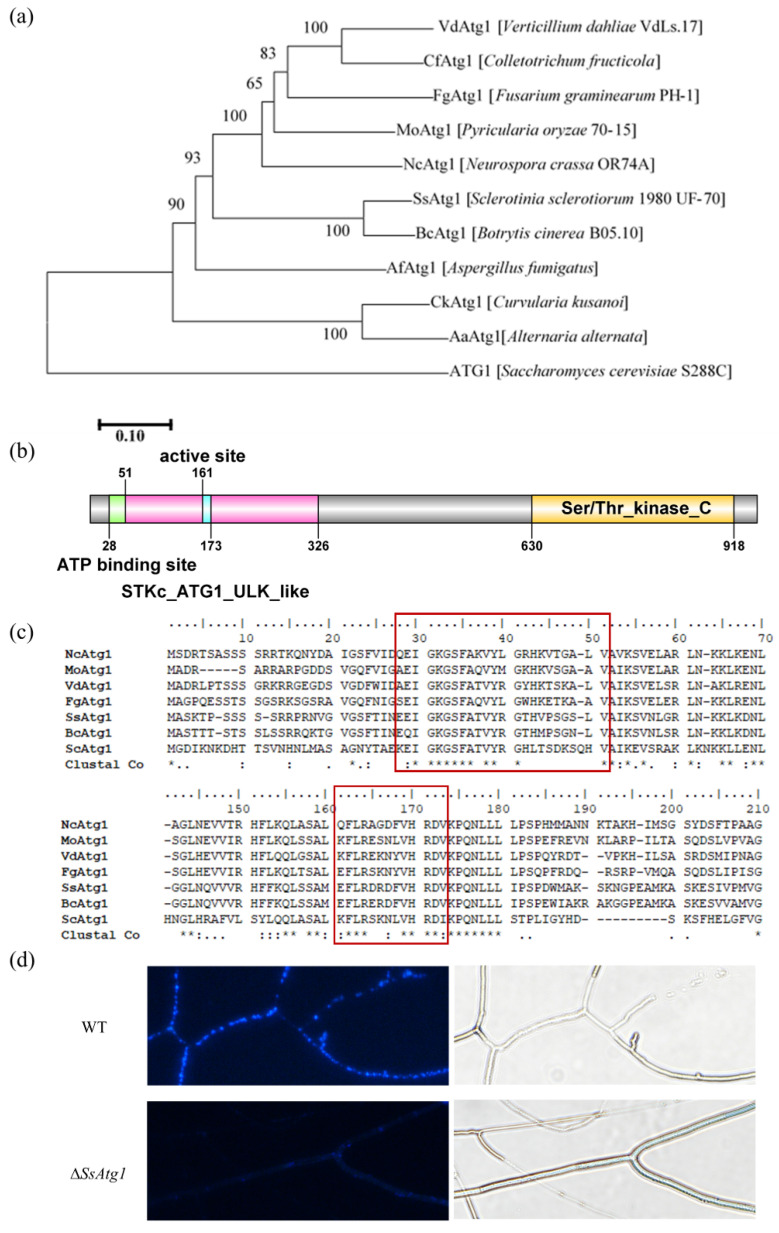
*SsAtg1* is a key gene activating autophagy in *S. sclerotiorum*. (**a**) Dendrogram of SsAtg1. Mega 7.0 was used to construct the phylogenetic tree. (**b**) Sequence information of SsAtg1. The conservation of the STKc_ATG1_ULK_like (cd14009) and Ser/Thr_kinase_C (IPR022708) domains was analyzed by InterPro (http://www.ebi.ac.uk/interpro, accessed on 18 February 2022) and GPS 2.0 was used for visualization. (**c**) Alignments of the ATP binding site and active site. Clustal W was used for alignment sequences and conserved amino acids residues are labeled as *. (**d**) Autophagy analysis of ∆*SsAtg1*. MDC is a fluorescent pigment used to detect autophagosome formation. Scale bar = 10 μm.

Phylogenetic analysis of the autophagy-related gene *SsAtg1* (*Sscle_12g087380*) with yeast and plant pathogenic fungi found that *SsAtg1* and *BcAtg1* (*B. cinerea*) were in the same clade (Figure 2a). Further sequence analysis revealed that *SsAtg1* encoded 951 amino acids which contain STKc_ATG1_ULK_like (CD14009) and Ser/Thr_kinase_C (IPR022708) conserved domains in the N-terminal and C-terminal, respectively. Position 28-51 contains a protein kinase ATP binding site (IPR017441) and a serine/threonine-protein kinase active site (IPR008271) at position 161–173 (Figure 2b). Analysis of ATG1 amino acid sequences from plant pathogenic fungi and yeast revealed that the active site of ATG1 was conserved at the serine/threonine-protein kinase active site (161–173) and the protein kinase ATP binding site (28-51). (Figure 2c). For the purpose of analyzing the biological functions of *SsAtg1* in *S. sclerotiorum*, *SsAtg1* was replaced with a hygromycin resistance gene by split-marker PCR, and UF/NLC37 and DR/NLC38 were used to verify that the *HYG* cassette replaced *SsAtg1* at the correct position. Primers SsAtg1 FP3/ SsAtg1 RP3 were used for PCR to verify the *SsAtg1* gene deletion (Figure S2a,b). Moreover, we investigated the expression of *SsAtg1* between the mutant and WT by RT-qPCR and found that the expression level was only 0.001 (Figure S2c), confirming that the *SsAtg1* mutant could be used in subsequent studies and split-marker PCR can be used for gene function studies in *S. sclerotiorum*. After nitrogen starvation induced autophagy, obvious autophagosome formation was observed in the wild type after treatment with MDC fluorescent dye, while no obvious autophagosomes were observed in ∆*SsAtg1* (Figure 2d).

### 3.3. SsAtg1 Is Indispensable for Mycelial Growth and Sclerotia Formation in S. sclerotiorum

**Figure 3:**
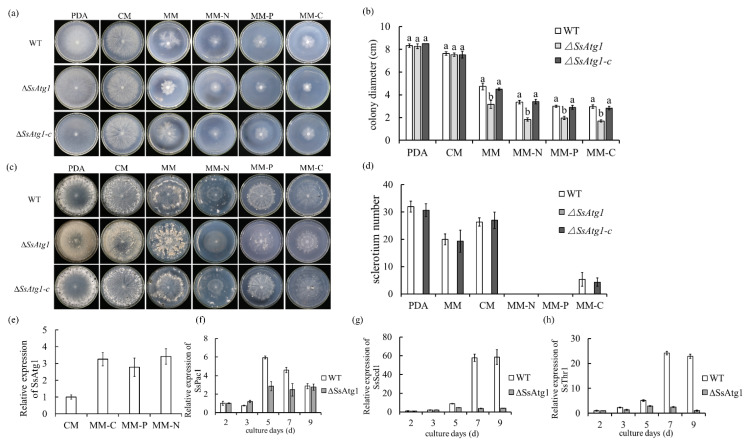
*SsAtg1* is required for sclerotium formation. (**a**,**b**) Colony morphology and colony diameter of ∆*SsAtg1* and control strains. Strains were inoculated on PDA, CM, MM, MM-N, MM-C and MM-P and colony diameters were measured by the cross method at 48 h. Error bars represent the standard deviations and the letters indicated significant differences (*p* < 0.05). (**c**,**d**) *SsAtg1* mutation affected sclerotia development. Colony morphology and sclerotia number were recorded after four weeks. Error bars represent mean standard deviation, and statistical significance of data was analyzed using SPSS software (*p* < 0.05). (**e**) The expression of *SsAtg1* was significantly increased in nutrient deficient medium. (**f**–**h**) Deficiency of *SsAtg1* affects the expression of key genes regulating sclerotia development.

Previously, we measured transcript accumulation of the *SsAtg1* (*Sscle_12g087380*) gene during the sclerotia formation stage and found that the expression level of *SsAtg1* in the early sclerotia formation and sclerotia development stages was significantly higher than that in the mycelia stage. This result further indicates that the autophagy pathway regulated by *SsAtg1* was important in the formation and development of sclerotia (Figure 1b). Phenotype observation of wild type, ∆*SsAtg1* and SsAtg1 complement transformant (∆*SsAtg1*-c) on PDA, MM and CM media showed that colony diameter of wild type, ∆*SsAtg1* and ∆*SsAtg1*-c displayed no significant difference on PDA and CM media, but colony diameter of ∆*SsAtg1* on MM, MM-N, MM-C and MM-P media was more restrained than that of wild type and ∆*SsAtg1*-c. These results indicated that *SsAtg1* affects vegetative growth and participates in nutrient utilization (Figure 3a,b).

Observations of sclerotia formation were conducted following four weeks of growth. Results showed that ∆*SsAtg1* could not form sclerotia on PDA, MM or CM media, and the colony color was darker than that of the wild type. On MM-N medium, which is a standard growth medium for induction of autophagy, the mycelial growth of ∆*SsAtg1* was restricted, and neither the wild type nor ∆*SsAtg1* could form sclerotia. These results further suggest that the initiation and mediation of autophagy are crucial to the initiation of sclerotia development and *SsAtg1* may affect secondary metabolism of *S. sclerotiorum* (Figure 3c,d).

Due to the nutritional deficiency phenotype of ∆*SsAtg1*, we measured the expression of *SsAtg1* under nutritional deficiency. The results showed that *SsAtg1* was significantly upregulated under MM-N, MM-C and MM-P which indicates that *SsAtg1* could regulate the growth of mycelia and the development of sclerotia in response to nutritional deficiency signals (Figure 3e). Subsequently, we explored relative expression of *SsPac1, SsScd1* and *SsThr1* during sclerotia development in wild type and ∆*SsAtg1*. Since ∆*SsAtg1* could not produce sclerotia, we compared it with wild type on the same culture days. *SsPac1* [36] is a homologous gene of transcription factor PacC, which regulated pH-sensitive gene expression. The expression of *SsPac1* was significantly increased at the early stage of sclerotia development, while the expression of *SsPac1* in ∆*SsAtg1* was lower than that of the wild type (Figure 3f). *SsScd1* and *SsThr1*, the melanin synthesis genes related to sclerotia formation, were up-regulated in the late stage of the wild-type sclerotia, namely, the sclerotia pigmentation stage, while there were no significant increases in the expression levels of *SsScd1* and *SsThr1* over time in the ∆*SsAtg1* culture (Figure 3g,h). The results indicate that the deficiency of *SsAtg1* not only affected the occurrence of autophagy, but also affected the essential metabolism process.

### 3.4. Deficiency of SsAtg1 Results in Abnormal Responses to Nutritional Stress 

**Figure 4:**
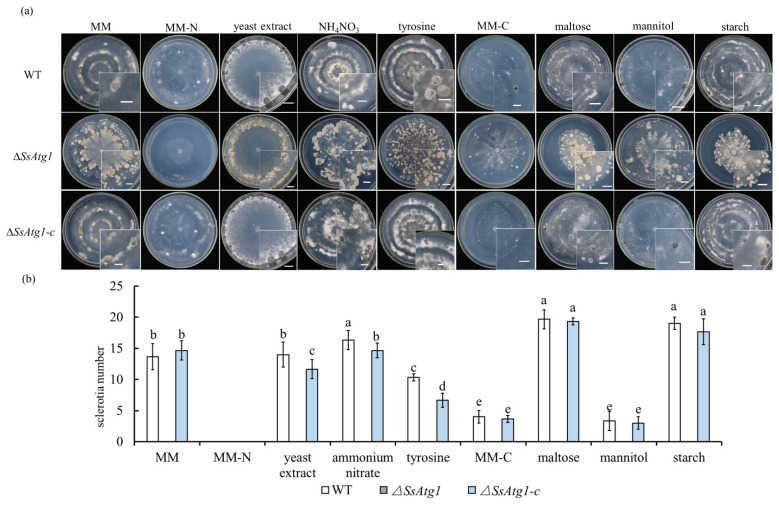
Role of *SsAtg1* in response to nutritional stress. (**a**) Colony morphology of wild type and ∆*SsAtg1* and the genetically complemented strain on different growth media. Strains were inoculated on MM replaced with indicated C sources and N sources. Scale bar = 0.5 cm. (**b**) Sclerotia number of ∆*SsAtg1* on different growth media. Colony morphology and sclerotia number were recorded after 4 weeks. The letters indicated significant differences (*p* < 0.05), error bars represent standard deviation from the mean.

Since the growth of ∆*SsAtg1* is significantly inhibited on MM medium, we explored the response of *SsAtg1* to nutritional stress by replacing C source and N source in MM medium. The results showed that with a complex organic nitrogen source, such as yeast extract, sclerotia are produced at the edge of the medium. The inorganic nitrogen source NH_4_NO_3_ had no significant effect on wild type sclerotia formation on MM. However, when tyrosine was added as a growth factor, the colonies of ∆*SsAtg1* were brown and dark mycelia aggregated on the surface of the medium; additionally, the size of wild-type sclerotia was significantly larger than that of other treatments (Figure 4a). In contrast to the lack of sclerotia in the absence of a N source, the wild type could still produce sclerotia in the absence of a C source, but the sclerotia diameter decreased (Figure 4a). When maltose and starch were used as C sources, more sclerotia were observed for the wild type, while colony growth of ∆*SsAtg1* was significantly inhibited (Figure 4a,b). Apparently, *SsAtg1* participates in and responds to nutritional stress.

### 3.5. ∆SsAtg1 Is Sensitive to Cell Wall Synthesis Inhibitors

**Figure 5:**
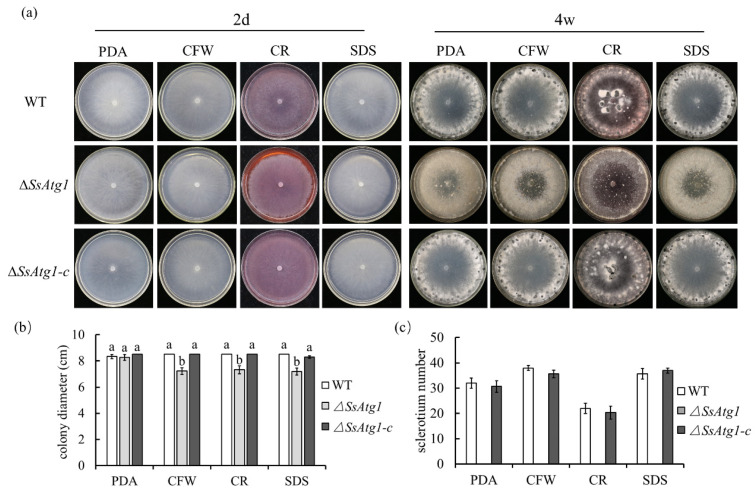
∆*SsAtg1* is sensitive to cell wall synthesis inhibitors. (**a**,**b**) Colony diameter and morphology of wild type, ∆*SsAtg1* and the genetically complemented strain. Strains were inoculated on PDA supplemented with CFW, CR and SDS and colony diameters were measured by the cross method at 48 h. (**c**) Quantification of sclerotia development in response to cell wall synthesis inhibitors. Colony morphology and sclerotia number were recorded after 4 weeks. The letters indicated significant differences (*p* < 0.05), error bars represent standard deviation from the mean.

In order to explore the influence of ∆*SsAtg1* on growth, cell wall inhibitors were added externally, the results showed that there was obvious differences in the growth rate in the presence of CFW, CR and SDS between ∆*SsAtg1* and wild type (Figure 5a,b). The wild type could develop normally and form sclerotium after adding cell wall inhibitors, but the sclerotia numbers were decreased after CR treatment. However, ∆*SsAtg1* could not form sclerotia after treatment with CFW, CR and SDS, and only loose aggregations of mycelia were observed (Figure 5a,c). The results suggest that *SsAtg1* plays a role in cell wall integrity in *S. sclerotiorum*.

### 3.6. Involvement of SsAtg1 in Pathogenicity and Compound Appressorium Formation

**Figure 6:**
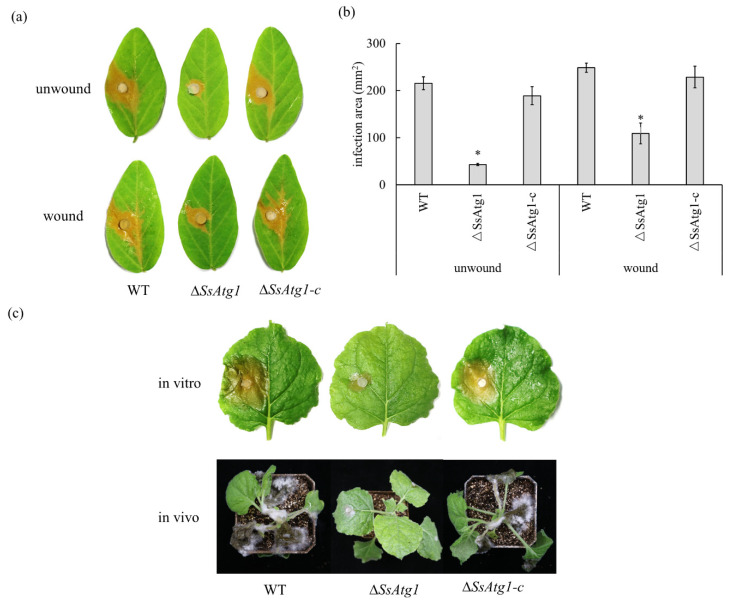
*SsAtg1* affects the virulence of *S. sclerotiorum*. (**a**) Pathogenicity of ∆*SsAtg1* on soybean leaves. (**b**) Quantification of the infection area. Image J was used to measure the infection area. (**c**) Pathogenicity of ∆*SsAtg1* on tobacco. Error bars represent the standard deviations and the asterisk indicated significant differences (*p* < 0.05).

Autophagy can be an important factor affecting pathogenicity. We measured the pathogenicity of ∆*SsAtg1* by wounded, unwounded, in vitro and in vivo inoculations. The virulence of ∆*SsAtg1* was significantly decreased both on wounded and unwounded soybean leaves (Figure 6a,b). Virulence of ∆*SsAtg1* on detached (in vitro) leaves and plants (in vivo) of tobacco was also observed. After inoculation, the leaves infected with the wild type were water-soaked and the whole plant wilted, however, the lesion area of the leaf inoculated with ∆*SsAtg1* was significantly reduced and the plant was intact (Figure 6c). These results confirm that *SsAtg1* affects the virulence of *S. sclerotiorum*.

**Figure 7:**
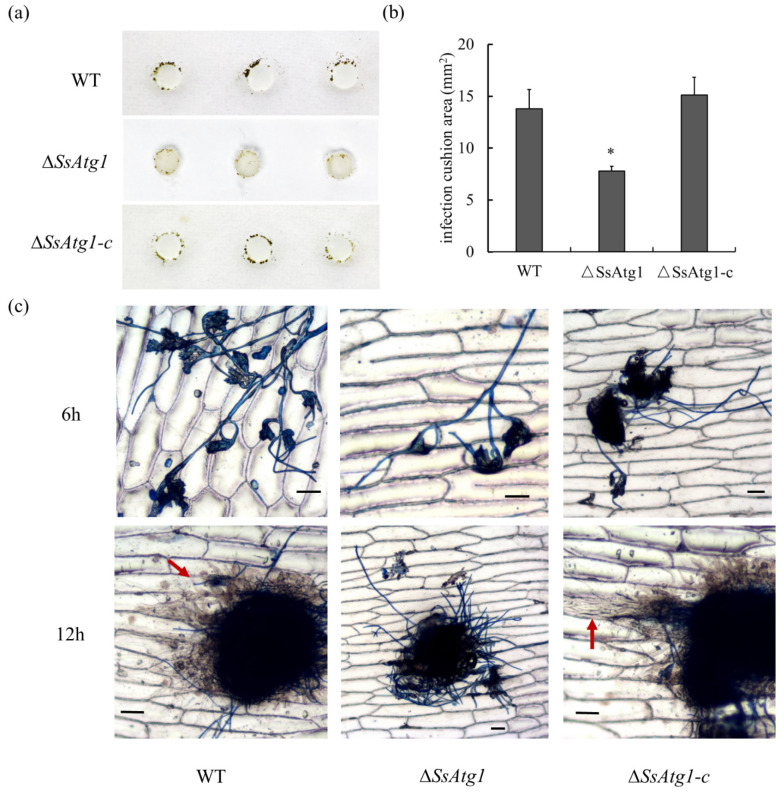
*SsAtg1* affected compound appressoria formation and penetration. (**a**,**b**) Compound appressoria of strains on hydrophobic interface. Image J was used for analysis of compound appressoria as: adjusted the image to 8 bit format, then selected the part of the compound appressoria by adjust-threshold, then using the area of the agar disk as the control to calculate the area of the compound appressoria. Error bars represent the standard deviations and the asterisk indicated significant differences (*p* < 0.05). (**c**) Compound appressoria formation on onion epidermal cells. The red arrow shows the invasion hypha which could not be stained by lactophenol cotton blue solution. Samples were staining by lactophenol cotton blue solution after 30 s, then washed with ddH_2_O and observed under a microscope. Scale bar = 50 μm.

Compound appressoria are an important factor of host plant infection by *S. sclerotiorum*. In order to further explore the factors of decreasing virulence of ∆*SsAtg1*, we measured the development and penetration of wild type and ∆*SsAtg1* compound appressoria. The results illustrated that the mass of ∆*SsAtg1* compound appressoria on an inert hydrophobic surface were decreased relative to the wild type (Figure 7a,b), but the morphology of the compound appressoria were normal (Figure S3a). Onion epidermis penetration experiments showed that wild type could penetrate the onion epidermis and form invasion hypha after 12 h post inoculation, however, the penetration efficiency of ∆*SsAtg1* was lower than that of the wild type (Figure 7c). The results indicate that *SsAtg1* affected the development and penetration of the compound appressoria.

### 3.7. SsAtg1 Disruption Caused Hypersensitivity to ROS

**Figure 8:**
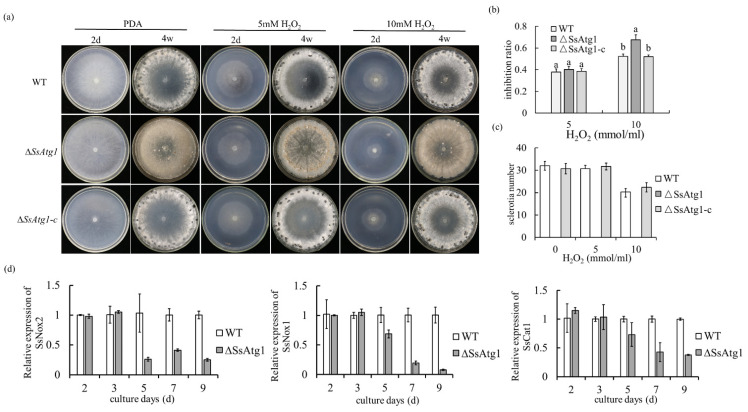
∆*SsAtg1* hypersensitivity to ROS. (**a**) Mycelia growth and morphology of the ∆*SsAtg1* under different concentrations of H_2_O_2_. (**b**) Inhibition rate analysis. Inhibition rate = (colony diameter of strain without H_2_O_2_ − colony diameter of strain with H_2_O_2_)/ colony diameter of strain without H_2_O_2_ × 100%. Error bars represent the standard deviations and the letters indicated significant differences (*p* < 0.05). (**c**) Statistics of sclerotia number. Significant difference in data by SPSS software (*p* < 0.05), error bars represent standard deviation. (**d**) Expression of reactive oxygen metabolism genes.

Redox dynamics are important in fungal growth, development and pathogenicity, and hyperoxidant states are the main drivers for microorganisms to enter into differentiation states [39]. Therefore, different concentrations of hydrogen peroxide were added to growth media and the growth inhibition rates of ∆*SsAtg1* and the wild type were examined. Under the treatment of 5 mmol H_2_O_2_ no differences were observed, but ∆*SsAtg1* was more inhibited than wild type under treatment with 10 mmol H_2_O_2_. This suggests that the autophagy pathway plays a role in maintaining the redox balance or responding to redox balance in *S. sclerotiorum* (Figure 8a,b). In terms of sclerotia formation, there was no sclerotia formation in ∆*SsAtg1*, but higher H_2_O_2_ treatment reduced the number of wild-type sclerotia, indicating that the redox state affects sclerotia formation of *S. sclerotiorum* (Figure 8a,c). Subsequently, we measured the expression levels of reactive oxygen metabolism genes *Ssnox1*, *Ssnox2* and *SsCat1* in wild type and ∆*SsAtg1*. Results showed that the expression levels of *Ssnox1*, *Ssnox2* and *SsCat1* in ∆*SsAtg1* were significantly lower than those in wild type during sclerotia formation (Figure 8d). This further indicates that *SsAtg1* participated in the response to oxidative stress and impacts the expression of ROS metabolism genes.

## 4. Discussion

Autophagy is a highly conserved process for degradation of aggregated and misfolded proteins and damaged organelles across eukaryotic species [14,15,40,41]. Autophagy uses the degraded materials for energy and cell structure reconstruction and is critical for maintaining cellular homeostasis [14,42]. Autophagy in filamentous fungi is related to pathogenicity, cell differentiation, nutrient balance and secondary metabolism [43,44,45,46]. Previous studies have shown that *SsAtg8* affects sclerotia formation, compound appressoria development and pathogenicity of *S. sclerotiorum* [9,10]. In this paper, the function of *SsAtg1* was studied. 

Autophagy-related protein Atg1 is the only protein kinase within the yeast ATG autophagy pathway. Disruption of *SsAtg1* affected the vegetative growth of *S. sclerotiorum*, and the colony growth of ∆*SsAtg1* was significantly restricted on MM and MM-N media, while the growth rate between ∆*SsAtg1* and wild type has no obvious difference in nutritionally adequate PDA and CM media. These results indicate that *SsAtg1* may affect the nutrient utilization of *S. sclerotiorum*, where a deficiency in SsAtg1 function whereby hyphae cannot provide growth substrates under conditions of nutrient deficiency is observed. 

Sclerotia play a central role in the asexual and sexual reproduction and disease cycle of *S. sclerotiorum* [5]. ∆*SsAtg1* cannot form sclerotia even after a prolonged duration in culture on PDA, CM and MM media, indicating that the autophagy pathway induced by *SsAtg1* is indispensable for sclerotia formation. In filamentous fungi, such as *M. oryzae*, *F. graminearum*, *B. cinerea* and *Aspergillus fumigatus*, loss of *atg1* function also affects the formation of asexual reproductive structures [23,24,47]. In addition, on N-starvation medium (MM-N) the wild type cannot form sclerotia, which further indicates that the induction and termination of autophagy are both crucial to the development of sclerotia. Furthermore, by replacing C and N sources in MM medium, we found that *SsAtg1* was a key gene for sclerotia formation in response to nutritional stress. Therefore, the relationship between autophagy and carbon metabolism in *S. sclerotiorum* deserves further study.

Compound appressoria and oxalic acid (OA) are important pathogenic factors for *S. sclerotiorum* [6]. Our pathogenicity analysis showed that the virulence of ∆*SsAtg1* significantly decreased relative to wild-type, subsequently, we analyzed the development and penetration of the compound appressoria in ∆*SsAtg1.* Results showed that the number of compound appressoria formed by ∆*SsAtg1* was lower than that in the wild type, simultaneously, compound appressoria penetration experiments on onion epidermis showed that the penetration efficiency of ∆*SsAtg1* was significantly decreased. In addition, the pH-indicator dye bromophenol blue was used to detect the in vitro accumulation of OA, and found that the mutant was the same as the wild type (Figure S3b). These results indicate that the influence of *SsAtg1* on the pathogenicity of *S. sclerotiorum* is multifactorial and related to compound appressoria and nutrient utilization but not OA accumulation. 

*S. sclerotiorum* secretes OA to trigger the host programmed cell death (PCD) which stimulates ROS production, and the enhanced ROS generation stimulates *S. sclerotiorum*-induced necrosis [48,49]. However, *SsAtg1* disruption caused hypersensitivity to ROS, which links autophagy with redox. In addition, the redox “climate” also plays a considerable role in fungal growth and development [39,50]. ∆*SsAtg1* is more sensitive to 10 mM hydrogen peroxide than the wild type; additionally, we determined that the expression levels of *Ssnox1*, *Ssnox2* and *SsCat1* genes related to reactive oxygen metabolism, were lower than those in wild type during sclerotia formation (Figure 8d). The results indicated that *SsAtg1* affects the expression of genes related to reactive oxygen species metabolism; moreover, ROS is important for sclerotia formation of *S. sclerotiorum*. 

In summary, we concluded that the *SsAtg1* gene of *S. sclerotiorum* is involved in response to nutritional stresses that govern mycelial growth and is essential for sclerotia formation, contributing to virulence and the development of compound appressoria.

## Data Availability

The data that support the findings of this study are available from the corresponding author upon reasonable request.

## Supplementary Materials

The following supporting information can be downloaded at: https://www.mdpi.com/article/10.3390/jof8121314/s1, Figure S1: COG and GO Function Analysis; Figure S2: Verification of ∆*SsAtg1*; Figure S3: Morphology of compound appressoria and the production of oxalic acid; Table S1: COG function classification; Table S2: DEG enrichment in GO annotation; Tables S3–S9: DEGs in transcriptome analysis; Table S10: Primer information.
