# Supplementary material for: The SsAtg1 Activating Autophagy Is Required for Sclerotia Formation and Pathogenicity in Sclerotinia sclerotiorum
Source: J Fungi (Basel). 2022 Dec 17;8(12):1314. doi: 10.3390/jof8121314 (PMC9787769; doi:10.3390/jof8121314)
Supplement: Supplementary file 1 [file jof-08-01314-s001.zip › Table S10.pdf]

Table S10 Primer information

Primers	Sequence（5’-3’）
SsAtg1F1	CTCCCGTGATTAGATTTG
SsAtg1R1	TCCTGTGTGAAATTGTTATCCGCTAAGGTTGGTGGACTTCGT
SsAtg1F2	GTCGTGACTGGGAAAACCCTGGCTCAGTCTCCCAACTCAAT
SsAtg1R2	ACAACCATAGGCACAATA
M13R	AGCGGATAACAATTTCA**C**ACAGGA
NLC37	GGATGCCTCCGCTCGAAGTA
M13F	CGCCAGGGTTTTCCCAGTCACGAC
NLC38	CGTTGCAAGACCTGCCTGAA
SsAtg1F3	AGCCTTGTTTACTGTTTGT
SsAtg1R3	GACTCGCAGCCATCTCAT
BD-SsAtg1F	GGATCCGTATGGCCTCCAAGACTCCTT
BD-SsAtg1R	CTGCAGTTAATGCGGTGGAGTATTGG
pYF11-SsAtg1F	GTAGGAACCCAATCTTCAAAATGGCCTCCAAGACTCCTTC
pYF11-SsAtg1R	AGCTCCTCGCCCTTGCTCACATGCGGTGGAGTATTGGCGA
QSsAtg1F	GCTTGCCGAGACTTTATGCG
QSsAtg1R	ATCGGCTCGTGCATCGTATT
actinF	GAATGTGTAAGGCCGGTTTCGC
actinR	CATCCCAGTTGGTGACGACACC
QSsscdF	ACTCGAATCCATCATCGCCC
QSsscdR	TCGCATGACTGTGTCCCTTC
QSsThrF	GAAATCGAGGGGCTCGGTAG
QSsThrR	ACCGGACATATTGGCAGCAA
QSsnox1F	TTTCTGGTCGGTTCACGGAG
QSsnox1R	CGAGACCTCGGGACAACAAA
QSsnox2F	AGCCAAACTCTACGACGGTG
QSsnox2R	CCAGTACCGATAAGGACCGC

QSspac1F	GATCCCCGTCGTCGTTACTC
QSspac1R	CAATTGACTCAGGGCGAGGT
