# Supplementary figures and images for: The SsAtg1 Activating Autophagy Is Required for Sclerotia Formation and Pathogenicity in Sclerotinia sclerotiorum
Source: J Fungi (Basel). 2022 Dec 17;8(12):1314. doi: 10.3390/jof8121314 (PMC9787769; doi:10.3390/jof8121314)

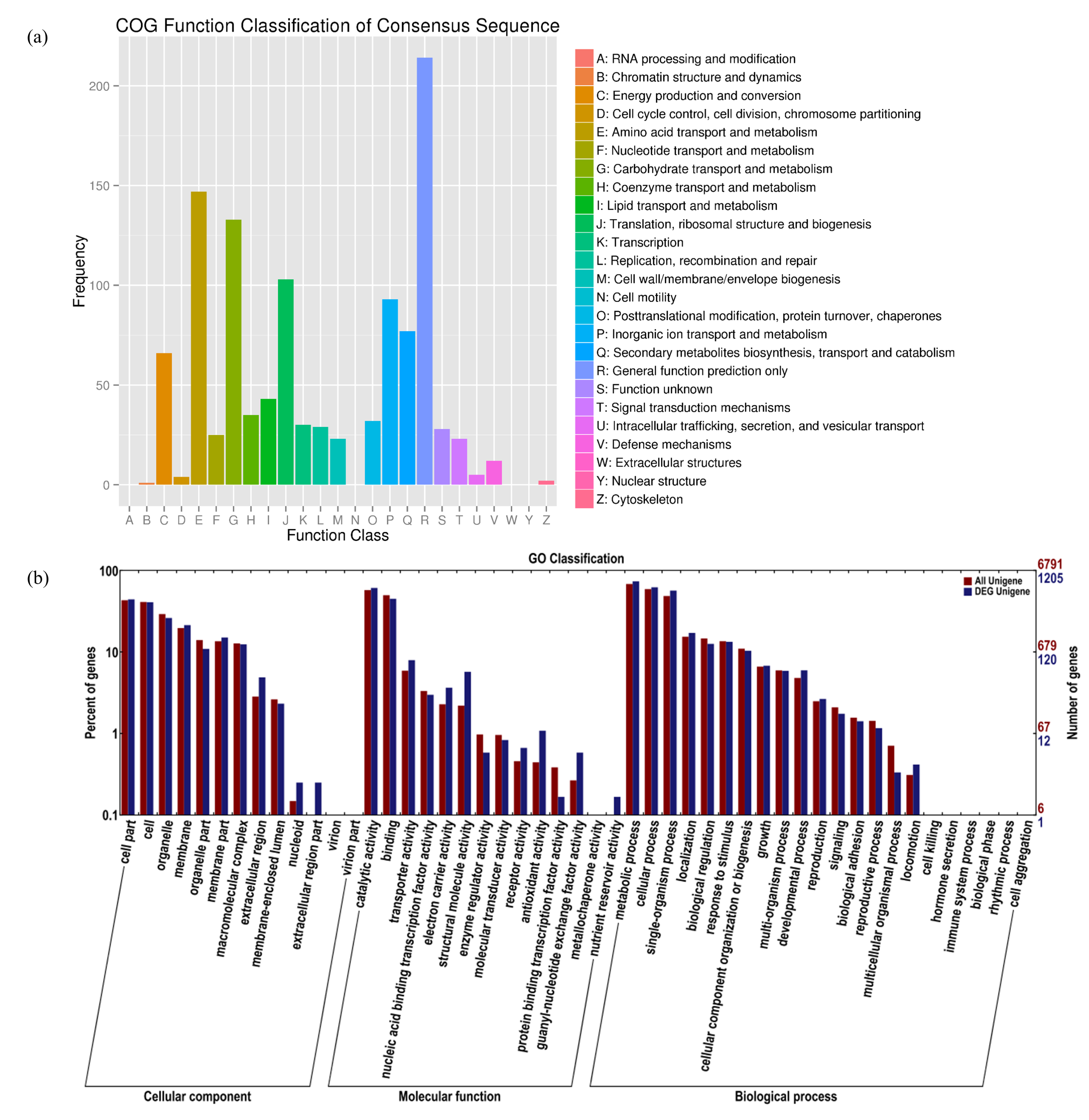

Supplement: Supplementary file 1 [file jof-08-01314-s001.zip › Figure S1.tif]

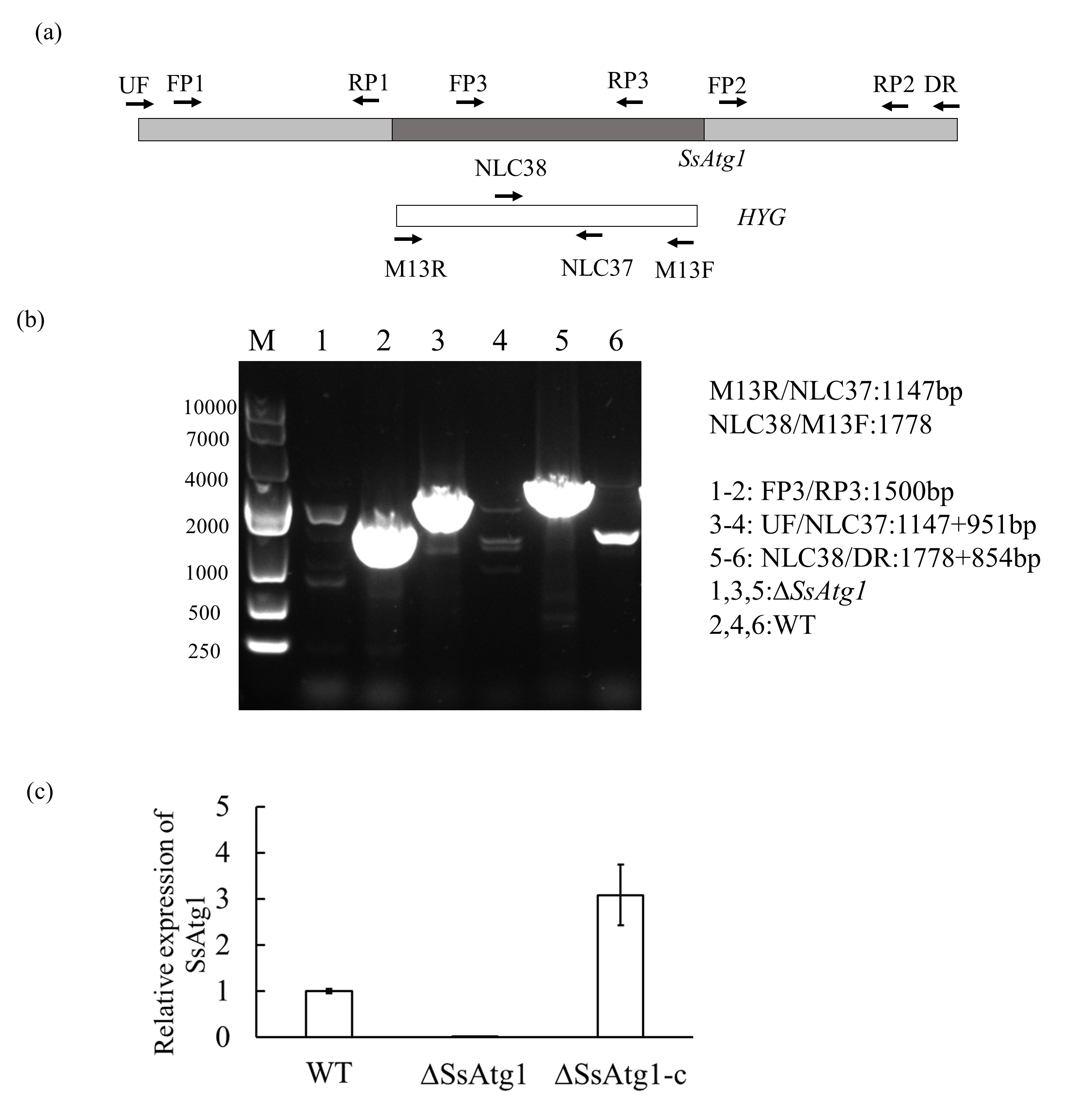

Supplement: Supplementary file 1 [file jof-08-01314-s001.zip › Figure S2.tif]

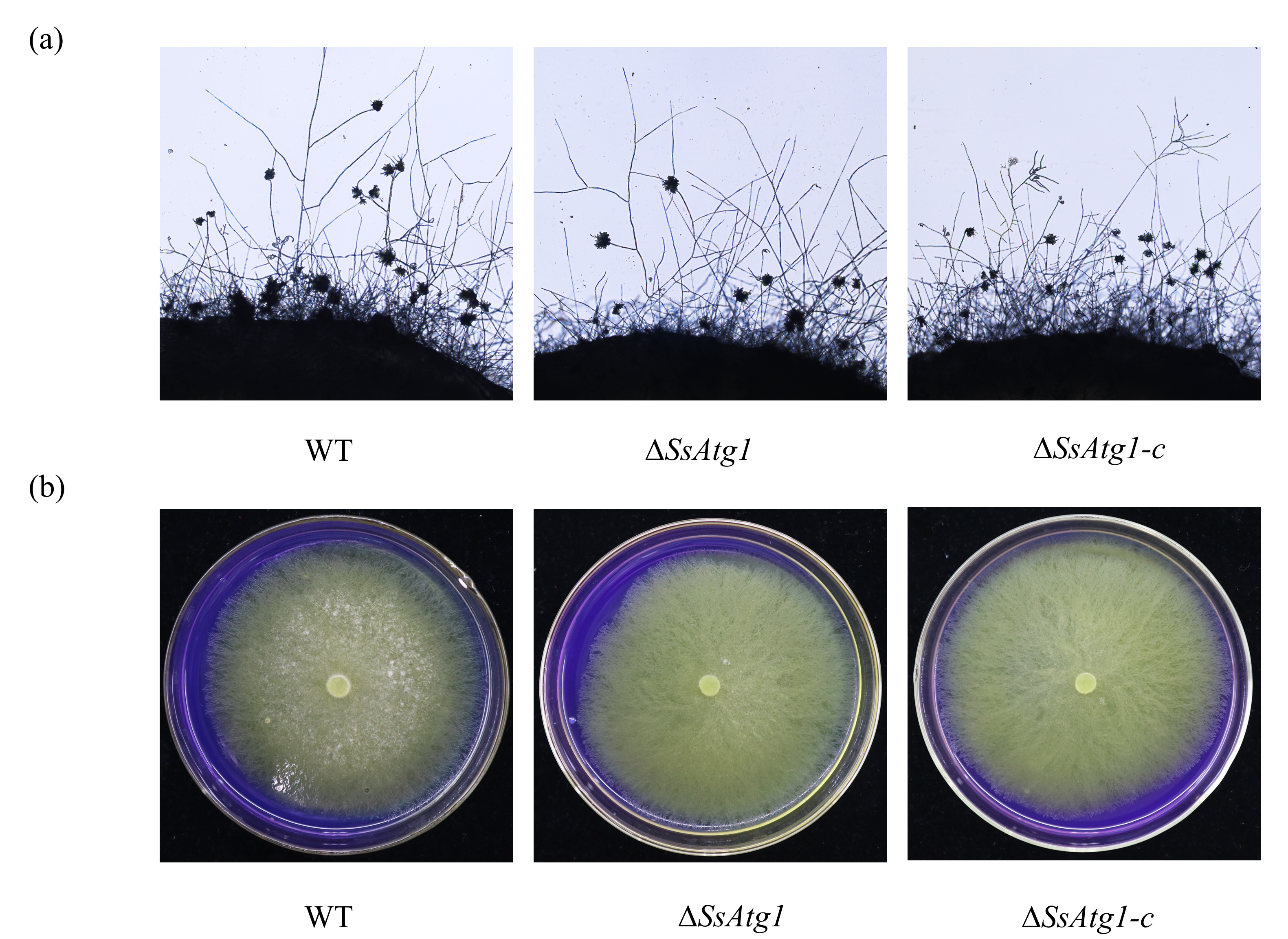

Supplement: Supplementary file 1 [file jof-08-01314-s001.zip › Figure S3.tif]
